# Pancreatic Aetiology for Massive Upper Gastrointestinal Haemorrhage in Pregnancy

**DOI:** 10.1155/2016/5491851

**Published:** 2016-02-29

**Authors:** Alexandra Zaborowski, Siun M. Walsh, Narayanasamy Ravi, John V. Reynolds

**Affiliations:** Department of Surgery, Trinity Centre, St. James's Hospital, James's Street, Dublin 8, Ireland

## Abstract

We present herein what we believe is the first reported case of massive upper gastrointestinal bleeding in pregnancy due to a pancreatic neuroendocrine tumour causing left sided portal hypertension. A 37-year-old 27-week pregnant female presented with massive haematemesis and melaena requiring transfusion of 10 units of red cell concentrate. Gastric varices were evident at endoscopy. An MRI revealed a large mass infiltrating the pancreatic tail and spleen with massive upper abdominal varix formation secondary to splenic vein invasion. A caesarean section was performed, followed by a radical en bloc partial pancreatectomy and splenectomy with resection of the fundus of the stomach and ligation of gastric and splenic varices. Her postoperative course was uncomplicated. Histology revealed a well differentiated grade 2 neuroendocrine tumour with final staging of T4N0. This case highlights an infrequently encountered cause of massive gastrointestinal bleeding. Diagnosis and management of pancreatic neuroendocrine tumours, due to their rarity and variable clinical presentation, can be challenging particularly in the setting of pregnancy where the wellbeing of a second patient must also be considered. A multidisciplinary approach with input from obstetricians and general surgeons is required when deciding optimum management, while also taking into account the patient's preferences.

## 1. Introduction

Pancreatic neuroendocrine tumours are rare tumours of the pancreas, associated with variable clinical presentations. Diagnosis antepartum results in significant additional therapeutic challenges and both risk of maternal disease progression and risk to the developing foetus must be considered when deciding optimum management. We present herein an unusual clinical presentation of a rare diagnosis and what we believe is the first reported case of massive upper gastrointestinal bleeding in pregnancy due to a pancreatic neuroendocrine tumour (NET) causing left sided portal hypertension.

## 2. Case Report

A 37-year-old woman, gravida 3, para 2+0, at 27-week gestation was transferred from a peripheral hospital with a three-day history of haematemesis and melaena requiring 10 units of red blood cells. An endoscopy prior to transfer showed numerous clots in the stomach but no bleeding source was identified. She had no significant past medical or family history and her pregnancy had been uncomplicated to date. Prenatal investigations had revealed a diagnosis of trisomy 21 and a congenital cardiac defect.

**Figure 1:**
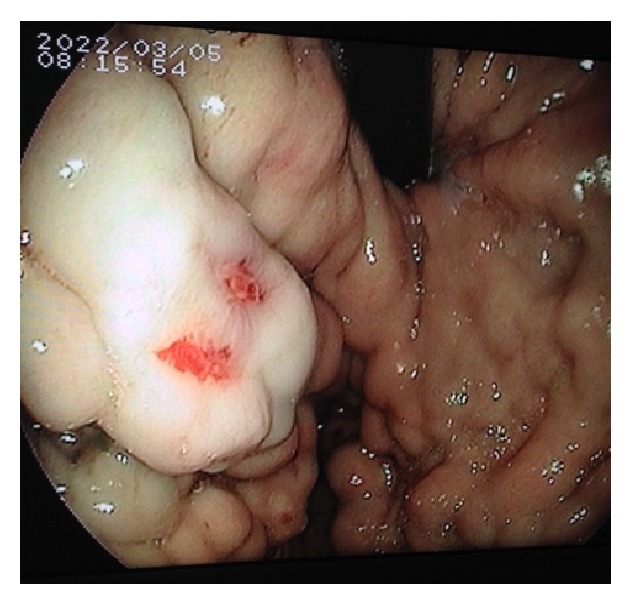
Upper gastrointestinal endoscopy showing extensive gastric varices.

**Figure 2:**
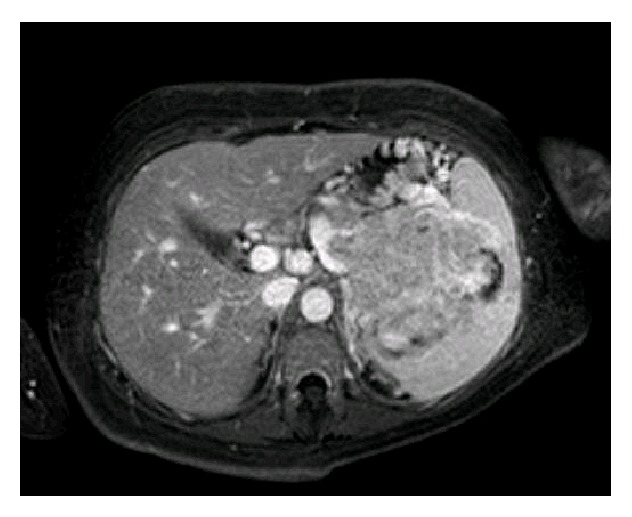
Magnetic resonance imaging of the abdomen was performed which revealed a 9 × 7 × 8 cm mass infiltrating the pancreatic tail and spleen along with massive upper abdominal varix formation secondary to splenic vein invasion.

On presentation, she was haemodynamically stable; her haemoglobin was 9.3 g/dL. Ultrasonography confirmed a viable pregnancy. Urgent upper gastrointestinal endoscopy revealed extensive gastric varices but no bleeding point and no oesophageal varices (Figure 1). Contrast enhanced magnetic resonance imaging (MRI) with diffusion-weighted sequences of the abdomen was subsequently performed which revealed a 9 × 7 × 8 cm mass infiltrating the pancreatic tail and spleen with splenic vein invasion, as well as consequent massive gastric varices along the greater curve and fundus (Figure 2). There was no evidence of liver metastases. Completion staging computed tomography (CT) of the thorax revealed no pulmonary metastases.

**Figure 3:**
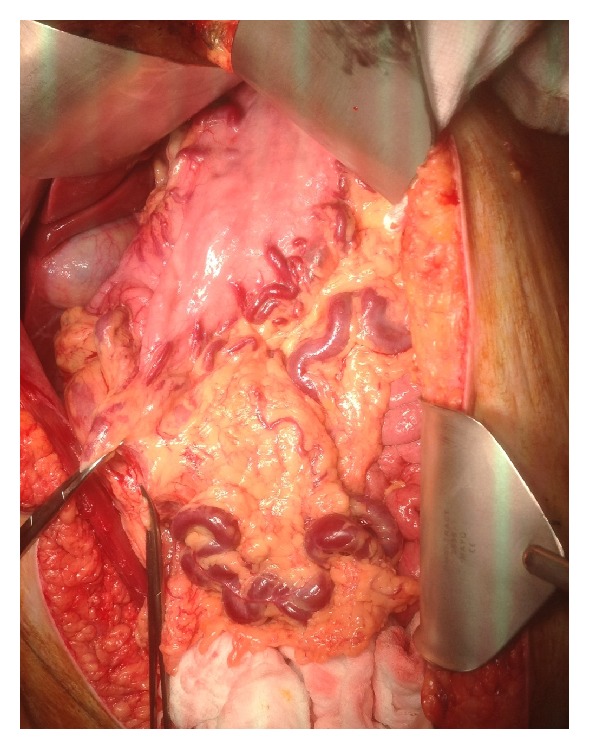
Laparotomy: extensive gastric varices.

Following consultation with the obstetrics team and the patient and her family, a plan was made to proceed with early surgical resection, given the high risk of rebleeding, with delivery of the foetus planned for the same day. She was given corticosteroids for 4 days and then underwent initial uncomplicated caesarean section followed by a radical en bloc partial pancreatectomy and splenectomy with resection of the fundus of the stomach and ligation of massive short gastric varices (Figure 3). Her postoperative course was uncomplicated and she was discharged home after two weeks. The neonate was transferred to a specialist neonatal unit and then discharged to the patient's local hospital for supportive care. He was discharged home well 6 weeks later.

Histology revealed a well differentiated grade 2 neuroendocrine tumour with a Ki67 of 10% and a mitotic rate 6 per 10 HPF. The surgical margins were free of tumour and 0/3 lymph nodes were involved. Final staging was T4N0. Her case was discussed at the gastrointestinal cancer multidisciplinary meeting and no further treatment was recommended.

Interval imaging with contrast enhanced CT thorax, abdomen, and pelvis at 3, 6, 9, and 18 months was performed with no evidence of disease recurrence or metastases. At 3 months postoperatively, an indium-111 octreotide single-photon emission CT scan (OctreoScan) demonstrated no evidence of metastases. At last follow-up and 24 months postoperatively the patient was well and free of disease.

## 3. Discussion

Pancreatic NETs are rare neoplasms that arise in the islet cells of the endocrine pancreas and account for less than 5% of all pancreatic tumours [1]. The biological profile and natural history of these tumours is variable; however the majority demonstrate a favourable prognosis compared to pancreatic adenocarcinoma [2]. They can be broadly classified into functioning and nonfunctioning based on hormonal secretion. Functioning NETs secrete a variety of hormones resulting in a myriad of clinical syndromes and account for approximately 25% [3]. The most common types are insulinomas, followed by gastrinomas, glucagonomas, VIPomas, and somatostatinomas. The remaining 75% of NETs are nonfunctional. These tumours may show immunohistochemical positivity for hormones but they do not secrete them [4]. Due to lack of associated hormonal symptoms nonfunctioning tumours tend to present at more advanced stages with signs and symptoms related to tumour burden or the presence of metastases. Nonfunctioning NETs can be further categorised into well or poorly differentiated based on degree of cellular differentiation and on mitotic count or Ki65 index [5]. The majority are sporadic but may also occur as part of the autosomal dominantly inherited syndrome Multiple Endocrine Neoplasia type 1, which is caused by inactivation of the tumour suppressor gene Menin located on chromosome 11q13 and associated with tumours of the anterior pituitary, parathyroids, and pancreas.

Endoscopic ultrasound, computed tomography, and magnetic resonance imaging are the primary modalities used to evaluate tumours of the pancreas, with MRI preferable in pregnancy due to high detection rate with the use of fat-suppressed T1 weighted images and lower foetal risk compared to computed tomography (CT) [6]. In addition the development of nuclear imaging modalities such as OctreoScan, ^18^FDG-PET/CT, and ^68^Gallium-PET/CT has further enhanced diagnostic accuracy.

Surgical resection is the cornerstone of treatment for localised disease [7, 8]. The optimum surgical approach or extent of lymphadenectomy however is uncertain. There is currently no consensus on the role of limited resection or enucleation and the prognostic significance of nodal metastases remains to be established [9]. Previous studies evaluating the impact of lymph node involvement on survival have shown conflicting results [9, 10]. Targeted adjuvant therapies are not routinely indicated where complete tumour removal with negative margins is achieved [11]. In contrast, the management of metastatic PNETs frequently involves combined modality therapy. The liver is a common site for metastases and the presence of hepatic disease does not necessarily preclude surgery. An aggressive surgical approach with resection of both the primary and the hepatic metastases where possible, achieves the best long-term results [12]. For patients with unresectable disease, local thermal ablative techniques, various forms of medical therapy, transcatheter embolisation or a combination of the aforementioned is recommended [13].

Given the unpredictable and variable natural history of PNETs development of metastases must be anticipated, particularly in this case, due to the large size of the tumour and extent of local invasion. In general guidelines recommend follow-up surveillance imaging with MRI or multiphasic CT 3 months after resection and annually thereafter. However, frequency and duration of surveillance may vary and patients with advanced tumours or unfavourable histological parameters may require follow-up imaging at shorter intervals [14].

Pancreatic disease is the most common aetiology of left sided portal hypertension. Splenic vein thrombosis is evident in up to 45% of patients with chronic pancreatitis, although most patients are asymptomatic [15, 16]. In this case, the presence of gastric varices despite normal liver function tests and ultrasound, and no risk factors or history of pancreatitis, demanded further investigations for a cause, with this rare diagnosis established.

We believe this case is the first pancreatic NET reported in pregnancy presenting with upper gastrointestinal bleeding from varices consequent of splenic vein invasion. There have been three documented cases of PNETs in pregnancy [17, 18]. These cases describe tumours diagnosed before 20-week gestation and resected antepartum with successful delivery at term in two of the three cases. Pancreatic tumours present a significant diagnostic and therapeutic challenge when diagnosed during pregnancy. Risk of maternal disease progression must be weighed against risk to the developing foetus and maternal/family preference taken into account [19]. The management option in a stable patient included delaying resection to a later stage of pregnancy; however in this case the magnitude of blood loss and its further threat, as well as the patient's own expressed wish, resulted in early surgery with a successful short term outcome.
